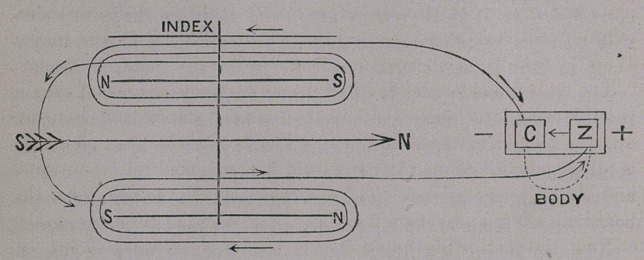# Neural Analysis

**Published:** 1882-04

**Authors:** B. Fincke

**Affiliations:** Brooklyn, N. Y.


### NEURAL ANALYSIS.

### BY THE ELECTRO-MAGNETIC METHOD.

B. Fincke, M. D., Brooklyn, N. Y.

The announcement of this new method in the *Hahnemannian Monthly*, June, 1881, has been noticed in The Homœopathic Physician, September, 1881, in a manner which is apt to mislead the reader to the conclusion that the instrument used labors under some errors which "should be corrected." There is nothing to be corrected. As it is, the errors are found to be on the critic's side, who wonders at the apparent impossible capability of the instrument to furnish a deflection of 245° from the point where the needle starts, viz.: 45° N. E. Now, anybody can see how the needle is deflected, after the introduction of the described galvanic current, beyond 90 degrees, and that ocular inspection, to which the critic and everybody is invited, settles the question.

Since then, the method has been improved, inasmuch as the operator himself is made the means of generating the electricity needed, besides conducting it through his body. For this purpose the galvanic battery is dispensed with altogether. Upon a small board, one-quarter inch thick, two inches by six, are fastened two squares of copper and zinc, of one-quarter inch size, about four inches apart, and they are connected with the wires of the galvanometer. As soon as the operator covers these two little plates with his thumbs, well wet with water, the needle is deflected to a greater or less degree, according to the potentiality of the nervous system. If two such plates are placed upon each other with a wet rag intervening, the needle, standing now at 60° Northeast, goes as far as 265° West, as the maximum deflection the instrument is capable of. The advantage in a comparison with the older method is in that the source of electricity will be constant, because at every operation the conditions are the same, viz.: the same surface of the heterogeneous metals, and the same amount of water applied by the covering skin of the thumbs, while, in the previous arrangement of a galvanic element, the current must, necessarily, gradually lose in force.

The double needle has been changed to a heavier one—No. 6, Milliner Milward—one and one-half inches long, which naturally takes the position of 60° Northeast, the point which has been found to yield the maximum of action. An extra circular measure has been put upon the dial, counting the regular stand of the needle at 60° Northeast as 1°, and extending from that point 270° West. The combination of two coils instead of using one is indeed, as the critic justly remarks, on account of the greater sensitiveness to electrical influence.

The following will convey the correct idea of how the instrument is built and operated (See diagram):

The two coils have their convolutions in the direction from left to right. They are placed one upon the other: the one, with 6,000 windings above; the other, with 5,700 below. The ends of wire coming from the innermost convolutions nearest to the neeedles are connected; those coming from the surface of the coils go to the poles of the galvanic element. In this way the outer end of the lower coil is connected with the positive, or zinc-pole, the outer end of the upper coil with the negative or copper-pole. As soon as the circuit is closed, the electric current runs from the positive pole, at the right hand, through the body to the negative pole, at the left hand; thence into the upper coil from outside till it reaches the windings around the upper needle; and then, in the opposite direction, it runs around the lower needle through the convolutions of the lower coil, returning through its outer end to the positive pole, at the right hand. Whilst this is going on, the needle is deflected to a certain degree, and, on opening the circuit, it returns in oscillations to its legitimate stand at 60° Northeast, in two to three minutes. The deflection of the needle is governed by Ampère's rule, which, in a popular way, he expressed thus: "The south pole of the needle is deflected towards that side from which the positive current appears to circulate, in the direction in which the index of the watch moves, *i.e.*, from left to right." Now, here, the positive current enters the upper coil first, and circulates around the needle in the direction from left to right, and the upper south pole, therefore, must be deflected towards you, if you stand on the west side. The next moment, the current enters the lower coil in the opposite direction, and therefore the lower south pole must be deflected in the opposite direction. But since, in the present arrangement, the needles within the coils cannot be observed, an index of indifferent nature is attached to the astatic system, above the coils, moving over a dial which indicates the deflection of the needle. And so it is that, when you cover the two plates with your wet thumbs, the zinc-plate with the right and the copper-plate with the left, the index parallel with the upper south pole pointing 60° Northeast, moves West. If the poles are reversed, it moves East.

The Galvanometer stands in the meridian upon a bracket near a window-casing, the wires run into two copper staples, a few inches apart, attached to the window sill. The board with the pole-plates is furnished with connecting wires which are hooked into the staples and always ready for use. The observer wets his thumbs, takes the board in hand, and covers the plates, and thus the circuit is closed.

Now, the interesting question presents itself: What is going on when the connecting link of the poles is the human body, which must be traversed at a length of 67 to 70 inches, from one thumb to another before any electricity can be developed at all? I had no occasion yet to experiment upon the dead body, but, judging from the fact that an exhausted, sleepy body gives very slight deflections, while a strong and vigorous one, especially under muscular and mental excitement, gives deflections which even can reach the maximum of action the instrument is capable of, it must be surmised that the current will not be generated and conducted by a dead body; a consideration which led my friend, Dr. John C. Robert, to the judicious remark that this method might be a safe means of deciding the certainty of death in dubious cases. Then the circumstance that the deflections vary in various observers, and in one observer at various times, and very distinctly immediately after taking medicine, points to the supposition that the current does not run indiscriminately the nearest way through the body, seeking its way through the multitude of moist tissues, or along the surface of the skin, as frictional electricity does, but that the medium is the nervous system.

From Volta's fundamental experiment we know that the origin of galvanism is not the chemical action, but the contact of heterogeneous metals. In our case, however, there is no such contact, because the metals are about 70 inches apart. Considering the galvanic element as used at first in this method, the water in which the metal-plates were immersed served as the medium for the exertion of their heterogeneous natures. There is no doubt that, if we put the plates, either of them alone, into the saucer with water, immediately an action takes place which tries to communicate the nature of the plate to the water. This looks more like a physical than a chemical action, and we might say that this action causes the electricity which appears when another plate of heterogeneous nature is put into the same vessel, and an arc applied, completing the circuit. For each of the plates may be considered as the cause and centre of an undulatory system, in which the circular waves of each are thrown around at first, until they intersect and interfere, reaching each other through these interferences, till the communication is established. These interferences, then, establish and equalize the electricity from the opposite poles. As soon as a conducting wire is applied to the negative pole and carried to the positive pole, the current of electricity forms out of the preliminary action, and the electricity is eliminated from the element, and in that manner, by giving way to new development, favors it, and furnishes power for work.

Now, here, in applying the thumbs to the plates, there is just as little reason to assign the origin of the electricity to chemical action because of a sheet of water mediating the contact between the plates and the skin. It leads back at once to Volta's fundamental experiment where the mere dry contact of zinc and copper makes the electrometer dilate. Here the wet contact of the plates with the skin produces the electricity which is taken up by the cutaneous nerves and carried up by undulatory motion to the centre to which they belong, analogous to the undulatory system in the simple element in water. This susception occurs on both sides, both of the systems on opposite sides meet and interfere in the centre, and there and then the positive pole preponderating gives the direction to the only then commencing current of electricity. It goes out of the body through the left thumb, circulates around and deflects the needle in the galvanometer, and returns to the positive pole. The body, therefore, plays a two-fold part: first, of generating the electricity in its contact with the metals, and, second, of conducting the thus generated electricity through the nervous system.

The difference between the generation of electricity by a special element for that purpose, and the interposition of the body in the negative pole as in the former method, and between that generation by means of direct contact of the body with the poles as in the present method, now acquires a significance of its own. For the latter will not only show the amount of conductibility of the nervous system, but also the capability of development, and will, therefore, prove a powerful instrument for physiological investigations in a new aspect.

But aside of the consideration of the developmental capacity and conductibility of the human system, important lessons may be drawn from the combination of this electro-magnetic method of Neural Analysis—the immortal discovery of Jaeger—with the chronoscopic which measures the time in which the deflections are reached, and it will be curious to see how the velocity of this electrical action in the nervous system will compare with the ingenious calculations of the velocity of nervous force of others.

In order to show what fine reaction the nervous system of man, and especially of woman, is capable of, the following observation upon Mrs. Dr. H. is given: She gave 127°, a second time 122°, then took one dose of *Calcarea carbonica* CM (Fincke); three minutes later the deflection was 27°, consequently 100° less than the first time; then, after about a quarter of an hour, 95°, and after an hour from the first observation, 170°.

Twenty years ago I had, as Dr. Adolph Lippe will remember, a small galvanometer of 100 yards wire, which he and afterwards Dr. Hering tried. The application was by copper- and zinc-handle, which were grasped by either hand. The effect was evident, but not satisfactory on account of its uncertainty of application to the body. After Jaeger came out with his wonderful discovery which forms an epoch in the history of medicine, I recommenced to work in the galvanic line, and now succeeded in finding the better application, by covering small plates with wet fingers. This seems to be the simplest and easiest way to experiment, and cuts off many sources of error which attach to the older methods.

In regard to the chronoscopic method of Neural Analysis, it may be permissible to add, that Prof. Dr. G. Jaeger, in Stuttgart, has already proved the power of the 4000th centesimal fluxion-potency of my preparation of *Natrum muriaticum* which "gave an increase of excitation of 55.4 per cent with very violent subjective sensations, which, after the inhalation, returned during 1½ minutes in attacks." *See Allgemeine Hom. Zeitung, Leipzig.* Vol. 103, p. 134. I have not the least doubt but that also by this method, with more sensitive observers, the hightest potencies will be shown to act.

*February 21st,* 1882.

## Figures and Tables

**Figure f1:**